# The influence of personality on the risk of myocardial infarction in UK Biobank cohort

**DOI:** 10.1038/s41598-022-10573-6

**Published:** 2022-04-25

**Authors:** Amelia D. Dahlén, Maud Miguet, Helgi B. Schiöth, Gull Rukh

**Affiliations:** Department of Surgical Sciences, Functional Pharmacology and Neuroscience, Uppsala University, Husargatan 3, BOX 593, 751 24 Uppsala, Sweden

**Keywords:** Psychology, Cardiology, Health care, Medical research, Risk factors

## Abstract

Personality is a strong determinant for several health-related behaviours and has been linked to the development of cardiovascular diseases. However, the reports of personality’s mediating role have been inconsistent with no data available from large population-based cohorts. The study aimed to create proxies for the Big Five personality traits, extraversion, agreeableness, conscientiousness, openness and neuroticism, to examine the longitudinal relationship between personality and myocardial infarction in the UK Biobank. The study sample comprised of 484,205 participants (55% female, 45% male, mean age 56.4 ± 8.1 years) from UK Biobank cohort with a mean follow-up of 7 years. The personality proxies sociability, warmth, diligence, curiosity and nervousness were created using self-reported data on psychological factors, mental health and social support, to match the facets of the Big Five traits. As neuroticism is the only Big Five personality trait available in the UK Biobank, it was included to validate the personality proxies. Myocardial infarction outcome information was collected from hospital records, death registries or was self-reported. Logistic regression and Cox proportional hazard regression were used to estimate odds ratio (OR) and hazard ratios (HR), respectively with 95% confidence intervals (CI) adjusted for demographics (age, sex, socioeconomic status, ethnicity), health-related factors (BMI, diabetes, systolic and diastolic blood pressure) and lifestyle factors (alcohol intake, smoking, and moderate-to-vigorous physical activity). Diligence was found to be significantly associated with lower prevalent myocardial infarction [OR: 0.87; (CI 0.84–0.89)] and lower incident myocardial infarction [HR: 0.88; (CI 0.85–0.92)]. Sociability was also protective against prevalent [OR: 0.89; (CI 0.87–0.92)] and incident [HR: 0.90; (CI 0.87–0.93)] myocardial infarction. Conversely, nervousness inferred a higher risk for both prevalent [OR: 1.10; (CI 1.08–1.12)] and incident [HR: 1.07; (CI 1.04–1.09)] myocardial infarction during follow-up. Sex-stratified analyses revealed that nervousness significantly increases the risk for incident myocardial infarction among women [HR: 1.13; (CI 1.08–1.19)] compared to men [HR: 1.05; (CI 1.02–1.08)]. By using our created proxies, we were able to investigate the impact of personality on the development of myocardial infarction. Persons with higher levels of diligence and sociability mimicking predominantly conscientiousness and extraversion personalities respectively are less likely to experience myocardial infarction, while personalities predominantly characterised by nervousness pose higher risk for developing myocardial infarction. These initial findings invite further validation of the use of the personality proxies in UK Biobank cohort.

## Introduction

Personality traits encompass relatively stable patterns of emotions and cognition which greatly influence daily behaviour throughout an individual’s life^1,2^. Personality is also closely intertwined with physiological responses to stress and has therefore been suggested to be involved in the progression of illnesses such as cardiovascular diseases (CVDs), which continue to be a leading cause of death worldwide^3,4^. Alongside the genetic and behavioural risk factors of CVDs, like high blood pressure, poor diet and insufficient physical activity, psychosocial factors in relation to cardiovascular health are becoming increasingly documented^5–7^. However, straightforward associations between specific personality characteristics and their potential to either increase or decrease the risk of developing CVDs are still lacking.

Since the late 1950s, several theories relating personality to the development of CVD have emerged^8^. Initially, aggressive and competitive individuals, denoted Type A personality, were considered at risk for coronary heart disease^8,9^. Later on, a more distressed profile with high negative affect, or Type D personality, was suggested to be overrepresented in cardiac patients and therefore a potential adverse trait^10^. Several taxonomies have been applied in the past to define the personality traits, however, the research in the last two decades has agreed on five broad personality dimensions known as the “Big Five”^11^. This model consists of five dimensions: extraversion versus introversion; agreeableness versus antagonism; conscientiousness; openness versus closedness to experience; and neuroticism versus emotional stability^12^. The highly influential Big Five model of personality has also been implicated in the development of CVD and may be a better predictor of health outcomes than the Type D personality^13,14^.

Higher scores on neuroticism and extraversion scales have been associated with increased risks of coronary heart disease^15,16^. In contrast, conscientiousness and openness have been suggested to be cardioprotective^15,17^. Personality may also interact with factors such as gender and socioeconomic status (SES) in mediating CVD^18,19^. For example, women with high neuroticism and low SES may have an elevated risk of CVD mortality, whereas high neuroticism and high SES may reduce the risk in women^19^. In other longitudinal work, neither neuroticism nor extraversion influenced the risk of CVD mortality to a significant degree^20,21^. However, using mortality as an endpoint inevitably rules out both prevalent and incident non-fatal cardiovascular events, resulting in fewer cases and weaker statistical power to detect slight changes in risk associated with psychological and behavioural dispositions^19–21^. In studies using non-fatal myocardial infarction (MI) as an endpoint, neuroticism was the only trait significantly associated with the incidence of MI^22^. On the other hand, a recent Mendelian randomization study found casual associations between neuroticism and the prevalence of atrial fibrillation, while no significant associations were detected with other CVDs such as myocardial infarction (MI) or coronary artery disease^23^. Thus, larger population-based studies are needed to clarify the mediating role of personality in development and progression of specific CVDs.

The primary aim of the present study was to utilise the power of large population-based UK Biobank cohort (UKB) to understand the effect of personality on MI. However, UKB only contains validated data on one of the five traits i.e., neuroticism. Thus, we aimed to identify phenotypes in the rich UKB dataset that can be used as proxies to represent all the Big Five personality traits. Then, to determine the predictive nature of the proxies, their association with MI was investigated both cross-sectionally and longitudinally. Neuroticism was used to validate the personality proxies. Elucidating how specific UKB-derived personality traits may influence the risk of disease, such as MI, will expand the possibilities of identifying at risk individuals and developing personality-focused interventions.

## Materials and methods

### Study population

**Figure 1:**
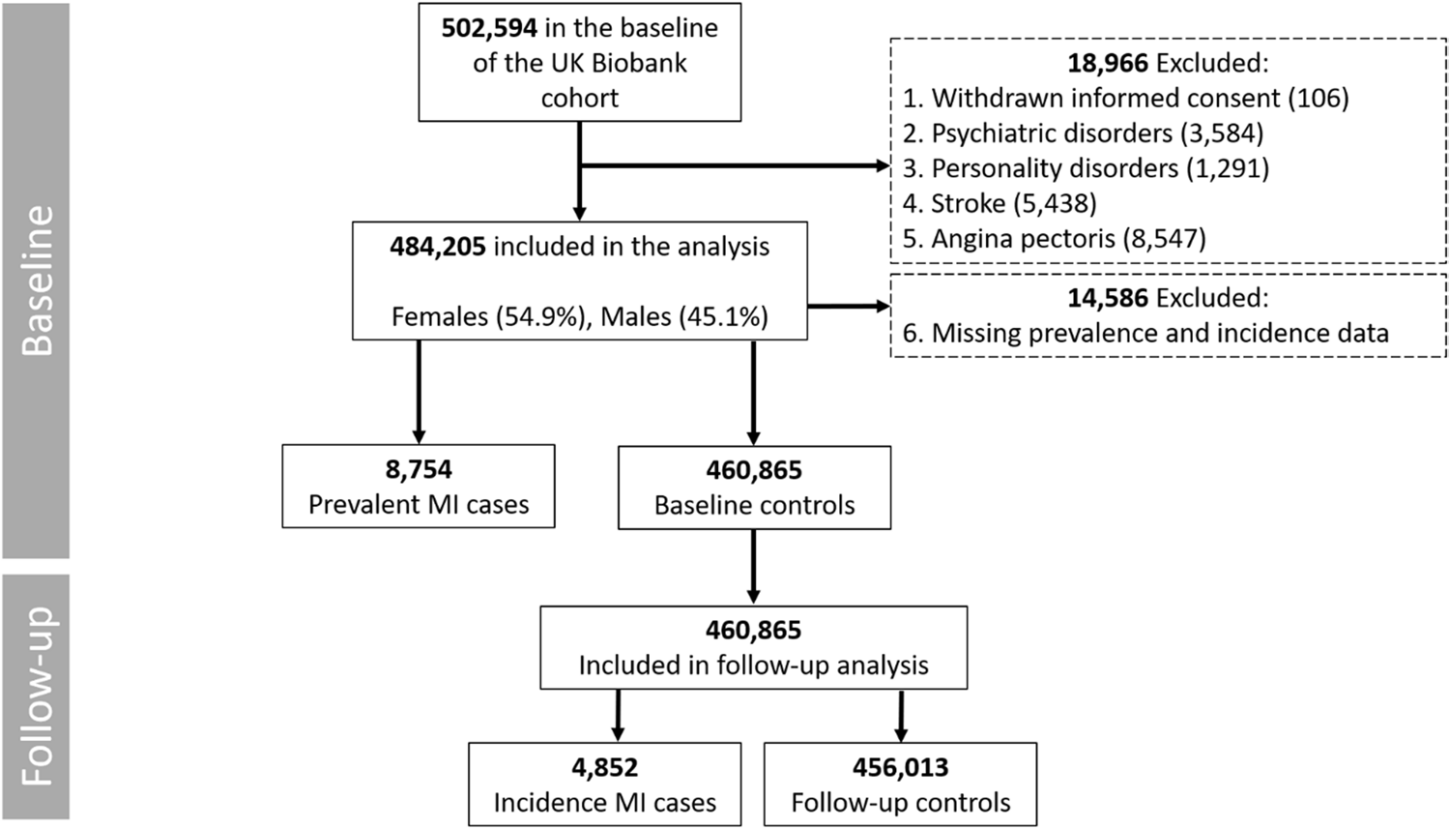
Study sample.

The UK Biobank (UKB), is a population-based prospective cohort study of 502,594 participants (aged 37–73 years), recruited between 2006 and 2010 from 22 assessment centers across the United Kingdom (UK)^24^. After the baseline assessment, the participants were followed up through their medical records, which include hospital records (Scottish Morbidity record and the National Care Record Service) and death registries (Office for National Statistics and Registrar General’s Office). All the participants who had withdrawn consent (n = 106), had history of psychiatric (n = 3584) and personality (n = 1291) disorders, stroke (n = 5438), angina pectoris (n = 8547) or had missing MI data (n = 14,586) were excluded. As personality traits may differentially affect different types of CVDs^25^, participants with history of stroke and angina pectoris were excluded as to not confound the potential associations between personality and MI. The detailed information regarding codes used to identify these conditions are provided in Supplementary Table S1. Thus, 484,205 participants were included in the present study (Fig. 1).

#### Ethical declarations

This study was carried out according to the Declaration of Helsinki. The UKB study was approved by the North West Multi-Centre Research and all participants provided written informed consent to participate in the UKB^24^. The present study was further approved by the Regional Ethics Committee of Uppsala, Sweden. Details of the UKB have been described elsewhere^26^.

### Study measures

#### Predictors

Creating psychological constructs is an intricate process, as their verity depends on the validity of their measurements^27^. Flawed measurements will make any statements about the construct uncertain, as it may not be exclusively measuring the intended phenomenon^27^. However, simplified constructs can be useful in large scale datasets like the UKB to identify predictors for the relevant outcomes. To address the lack of a specific validated scale within the UKB, prior studies have used the available self-reported information to create scales resembling psychological constructs, e.g., loneliness and social isolation^28,29^. These one- to three-point scales have been successful in replicating associations found using the corresponding validated constructs across a range of outcomes, such as MI, self-harm and excess mortality, with the added benefits of high statistical power and longitudinal analyses^28–31^.

In a similar manner as the studies presented above, the current UKB proxies for personality were created using data from touchscreen questionnaires on psychological factors, mental health and social support completed by the participants at the baseline assessment. A validated personality scale, the Big Five Inventory^12^, was carefully consulted and thereafter similar variables from the UKB were identified to best match the facets of the Big Five Inventory measure. Reverse coding was used for some items to construct the proxies. The a priori selected questions for each personality trait resulted in five scales scored between zero and four (sociability, diligence and curiosity) and zero and five (warmth and nervousness) (Table 1). As the proxies have not been externally validated, it is important to interpret the results with caution.

**Table 1:** Big Five proxies derived from UK Biobank variables.

Personality proxy	Facets of *BFI	UK Biobank questions	Code
SOCIABILITY (Extraversion vs Introversion)	Gregariousness (sociable) Assertiveness (forceful) Positive emotions (enthusiastic) Activity (energetic) Excitement-seeking (adventurous) Warmth (outgoing)	Frequency of friend/family visit (> once a month)	1031
		Guilty feelings (no)	2030
		Frequency of tiredness/lethargy in last 2 weeks (not at all)	2080
		Leisure/social activities	6160
WARMTH (Agreeableness vs Antagonism)	Trust (forgiving) Straightforwardness (not demanding) Altruism (warm) Tender-mindedness (sympathetic) Modesty (not show-off) Compliance (not stubborn)	Able to confide to (> once a month)	2110
		Irritability (no)	1940
		Mood swings (no)	1920
		Tense / 'highly strung' (no)	1990
		Nervous feelings (no)	1970
DILIGENCE (Conscientiousness vs lack of direction)	Competence (efficient) Achievement striving (thorough) Self-discipline (not lazy) Deliberation (not impulsive) Dutifulness (not careless) Order (organized)	Frequency of enthusiasm/disinterest in last 2 weeks (not at all)	2060
		Fed-up feelings (no)	1960
		Risk taking (no)	2040
		Worry too long after embarrassment (yes)	2000
CURIOSITY (Openness vs closedness to experience)	Ideas (curious) Actions (wide interests) Feelings (excitable) Values (unconventional) Aesthetics (artistic) Fantasy (imaginative)	Loneliness, isolation (no)	2020
		Suffer from 'nerves' (no)	2010
		Frequency of tenseness/restlessness in last 2 weeks (> several days)	2070
		Risk taking (yes)	2040
NERVOUSNESS (Neuroticism)	Anxiety (tense) Angry hostility (irritable) Depression (not contented) Self-consciousness (shy) Impulsiveness (moody) Vulnerability (not self-confident)	Tense / 'highly strung' (yes)	1990
		Irritability (yes)	1940
		Frequency of enthusiasm/disinterest in last 2 weeks (> several days)	2060
		Mood swings (yes)	1920
		Sensitivity/hurt feelings (yes)	1950

*Big Five Inventory (BFI) (John and Srivastava^12^).

“Sociability” scale was constructed using four questions: (1) "How often do you visit friends or family or have them visit you?" (1 point for visit frequency ≥ about once a month), (2) "Are you often troubled by feelings of guilt?" (1 point for no), (3) "Over the past 2 weeks, how often have you felt tired or had little energy?" (1 point for not at all) and (4) "Which of the following do you attend once a week or more often?" (1 point for sports club or gym, pub or social club, religious group, adult education class or other group activity).

“Warmth” scale was constructed using five questions: (1) "How often are you able to confide in someone close to you?" (1 point for confiding frequency ≥ about once a month), (2) "Are you an irritable person?" (1 point for no), (3) "Does your mood often go up and down?" (1 point for no), (4) "Would you call yourself tense or 'highly strung'?" (1 point for no) and (5) "Would you call yourself a nervous person?" (1 point for no).

“Diligence” scale was constructed using four questions: (1)"Over the past two weeks, how often have you had little interest or pleasure in doing things?" (1 point for not at all), (2) "Do you often feel 'fed-up'?" (1 point for no), (3) "Would you describe yourself as someone who takes risks?" (1 point for no) and (4) "Do you worry too long after an embarrassing experience?" (1 point for yes).

“Curiosity” scale was constructed using four questions: (1) "Do you often feel lonely?" (1 point for no), (2) "Do you suffer from 'nerves'?" (1 point for no), (3) "Over the past two weeks, how often have you felt tense, fidgety or restless?" (1 point for ≤ nearly every day) and (4) "Would you describe yourself as someone who takes risks?" (1 point for yes).

“Nervousness” scale was constructed using five questions: (1) "Would you call yourself tense or 'highly strung'?" (1 point for yes), (2) "Are you an irritable person?" (1 point for yes), (3) "Over the past two weeks, how often have you had little interest or pleasure in doing things?" (1 point for ≤ nearly every day), (4) "Does your mood often go up and down?" (1 point for yes) and (5) "Are your feelings easily hurt?" (1 point for yes).

The UKB Neuroticism score was derived by Smith et al.^32^. The score was based on twelve neurotic behavioural domains from the Eysenck Personality questionnaire (EPQ-N), and ranges from zero to twelve. A higher score indicates a higher degree of neurotic behaviour.

#### Study endpoints

MI events were identified using algorithmically defined outcome variables, developed by the UKB adjudication committee, and created through linkage of hospital admission data from England, Wales and Scotland, national death registries and self-reported data. The variables contained information about the date of the first-known MI and have been used elsewhere^33^. In these variables, MI events were recorded by using the following International Classification of Disease (ICD)-10 codes: MI: I21, I22, I23, I24.1 and I25.2; as well as by the following ICD-9 codes from the Scottish Morbidity record: MI: 410, 411, 412.X and 429.79. For incident MI events, follow-up time started at the date participants attended the baseline assessment until March 1, 2016 or until the occurrence of the first MI event.

### Study covariates

The covariates taken into account for their potential confounding effects regarding MI, were divided into three categories; demographic characteristics, health-related measurements and lifestyle factors. The association of all the covariates with MI is shown in Supplementary Table S2.

#### Demographics

Baseline characteristics such as sex, age, ethnicity, and socioeconomic status were collected during the initial assessment. Ethnicity was defined as white (white, British white, Irish, any other white background) versus other ethnicities, such as black, Asian, Pakistani, Indian, Mixed, Chinese and other ethnic groups. Townsend Deprivation Index (TDI), calculated based on participants’ postal code and information from the national census output area, was used as a proxy for socioeconomic status.

#### Health related measurements

Health related measurements were also recorded during the baseline visit. Weight was measured using Tanita BC-418MA body composition analyzer and height with Seca 202 height measure, and they were used to calculate the body mass index (BMI; kg/m^2^). Systolic blood pressure (SBP) and diastolic blood pressure (DBP) were measured, using Omron HEM-705 IT digital blood pressure monitor, twice with 1-min interval between the two measurements. The average SBP and DBP were calculated and used in the analyses. Diabetes was defined using the ICD-10 primary/main diagnosis codes from hospital inpatient records: E10–E14.

#### Lifestyle factors

Lifestyle-related information was collected using touch screen questionnaires. Smoking status was divided into three groups (never, previous and current), while alcohol intake frequency was divided into six groups (daily or almost daily, three or four times a week, once or twice a week, one to three times a month, special occasions only, never). Moderate-to-vigorous physical activity (MVPA) score level was derived by questions aimed at weekly frequency and daily duration (in minutes) of moderate physical activity and vigorous physical activity. The variables were handled according to the guidelines for data processing and analysis of the international physical activity questionnaire (IPAQ)^34^ and they were used to calculate the metabolic equivalent per time (MET) score of MVPA based on the following formula: MVPA score (MET-min/week) = [(Number of days/week of moderate physical activity 10 + minutes × Duration of moderate physical activity × 4.0) + (Number of days/week of vigorous physical activity 10 + minutes × Duration of vigorous physical activity × 8.0)]^35^. The MVPA score (MET-minutes/week) variable was further divided into automatically generated quantiles by ranking all cases with 1 in the lowest value, resulting in the formation of a ranked categorical variable with 4 groups. For all variables, the responses “do not know” and “prefer not to answer” were coded as missing values.

### Statistical analysis

Descriptive statistics for continuous variables are presented as a mean (± standard deviation (SD) of the mean) and for categorical variables as number (percentage (%)). Associations between the personality proxies and, prevalent and incident MI were examined using logistic regression and Cox proportional hazard regression, respectively. The results are presented as odds ratios (OR) or hazard ratios (HR) and 95% confidence intervals (CI). To assess the potential confounding or mediating effects of the selected covariates on the prevalence and incidence of MI, three models were implemented for each personality proxy to sequentially adjust for all covariates. Model I (basic model) included demographic characteristics (sex, age, TDI and ethnic background). Model II included all covariates in model I plus BMI, SBP, DBP and diabetes. Model III (or fully adjusted model) included all covariates in model II, plus lifestyle factors (alcohol intake frequency, smoking status, and physical activity). Participants without an MI event were used as the reference group in all analyses. All statistical analyses were performed using SPSS software (IBM SPSS Statistics version 26) and horizontal forest plots were created using GraphPad Prism version 9.1.2 (1992–2021 GraphPad Software, LLC, US; http://www.graphpad.com). To account for multiple testing for two outcomes (prevalent and incident MI), Bonferroni correction was applied and a p < 0.025 (i.e., 0.05/2) was considered significant in all analyses.

### Ethics approval and consent to participate

The UKB study was approved by the North West Multi-Centre Research Ethics Committee, the Regional Ethics Committee of Uppsala, Sweden and all participants provided written informed consent to participate in UKB study (approved UKB application no. 25308 and 30172).

## Results

Based on the exclusion criteria in Fig. 1, 484,205 participants were included in the present study, of which 8,754 participants (1.81%) had an MI event before baseline assessment (prevalent cases) and 14,586 participants had missing prevalence and incidence data and were excluded. The remaining, 460,865 participants were included in the longitudinal analysis and during the median follow-up time of 7.00 years (Q1: 6.0; Q4: 9.0), 4,852 (1.05%) participants had MI events (incident cases) (Fig. 1).

The participant sample consisted of 54.9% women and 45.1% men with a mean age of 56.4 (± 8.1) years. Men were overrepresented in both prevalent MI (80.1%) and incident MI (72.0%) cases. Moreover, men reported a more frequent intake of alcohol compared to the women (Table 2).

**Table 2:** Characteristics of study participants.

Characteristics	Women (n = 265 841) (54.9%)	Men (n = 218 364) (45.1%)
Age, years	56.3 (8.0)	56.6 (8.2)
**Ethnicity, n (%)**		
White	242 550 (91.2)	200 205 (91.7)
Other	23 291 (8.8)	18 159 (8.3)
**Blood pressure, mm Hg**		
Systolic	137.2 (20.3)	142.7 (18.5)
Diastolic	80.7 (10.6)	84.1 (10.5)
**Smoking status, n (%)**		
Never	158 254 (59.5)	107 589 (49.3)
Previous	82 926 (31.2)	83 872 (38.0)
Current	23 218 (8.7)	26 584 (12.2)
**Alcohol consumption, n (%)**		
Never	24 753 (9.3)	13 356 (6.1)
Twice or less per week	142 679 (53.7)	91 708 (42.0)
At least three times per week	97 719 (36.7)	112 622 (51.6)
**Body mass index, kg/m**^**2**^	27.1 (5.2)	27.8 (4.2)
**Socioeconomic status, score**		
Mean Townsend deprivation index score	− 1.35 (3.0)	− 1.29 (3.1)
**Diabetes, n (%)**	616 (0.2)	1 075 (0.5)
**Drug use, n (%)**		
Antihypertensive drugs	32 (0.01)	75 (0.034)
Lipid-lowering drugs	9 (0.003)	16 (0.007)
**Prevalent myocardial infarction, n (% of total MIs)**	1 738 (19.9)	7 016 (80.1)
**Incident myocardial infarction, n (% of total MIs)**	1 357 (28.0)	3 495 (72.0)

Values are presented as mean and standard deviation (SD) in brackets for continuous variables and as number of participants (n) and percentage (%) for categorical variables.

### Association of personality trait proxies with prevalent MI

**Figure 2:**
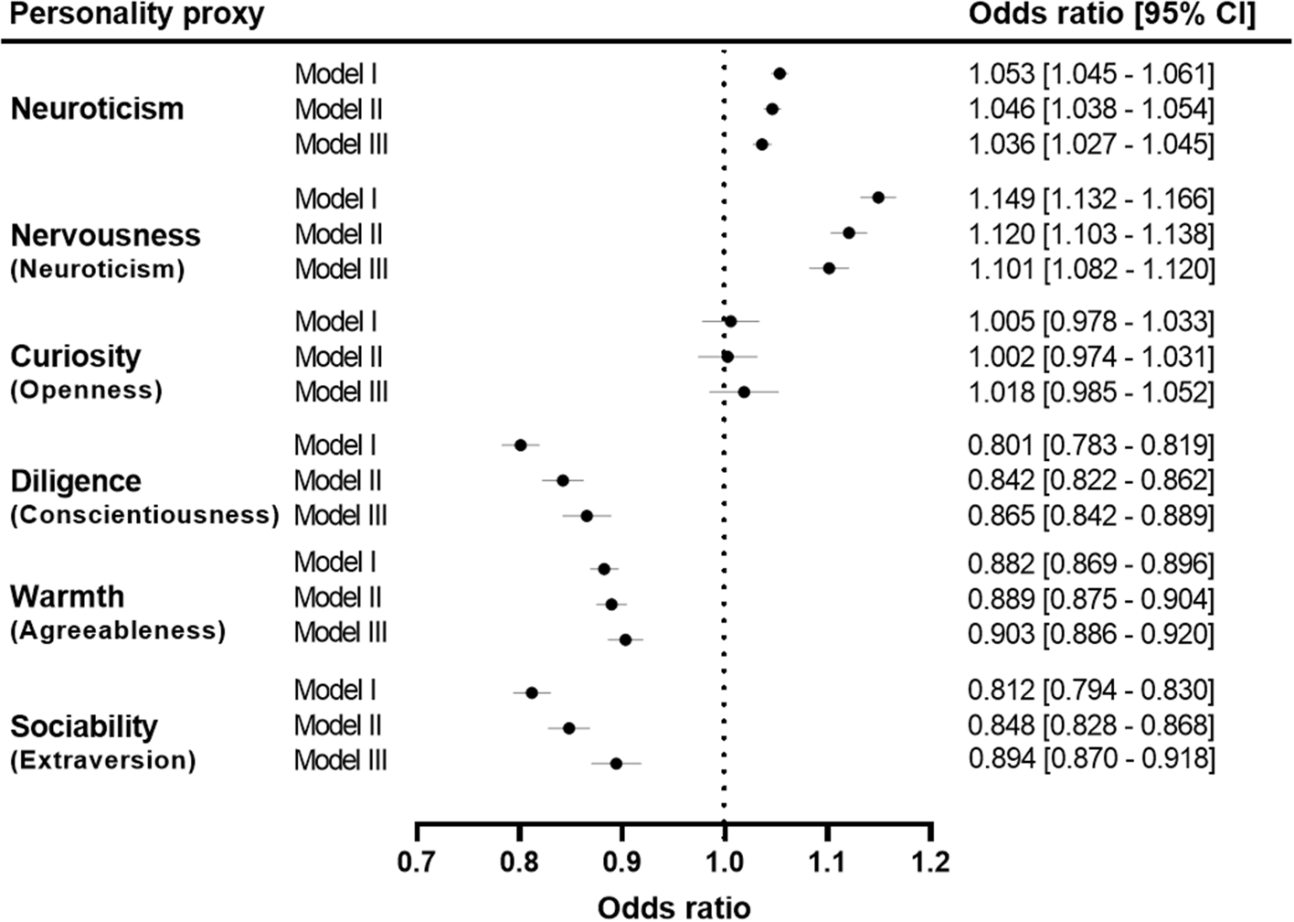
Association of neuroticism and personality trait proxies with prevalent myocardial infarction. Labels in small brackets represent original Big Five personality trait corresponding to each personality trait proxy. Model I: analyses were adjusted for demographic factors (sex, age, Townsend Deprivation Index and ethnic background). Model II: analyses were adjusted for health-related measures (body mass index, systolic blood pressure, diastolic blood pressure and diabetes) in addition to demographic factors. Model III (or fully adjusted model): analyses were adjusted for lifestyle factors (alcohol intake frequency, smoking status, and physical activity) in addition to demographic factors and health-related measures. The plot was created using GraphPad Prism (9.1.2).

Model I, adjusted for demographic characteristics, revealed that neuroticism [OR: 1.05; (95% confidence interval (CI) 1.05–1.06); (P = 2.15 × 10^–42^) and its proxy nervousness [OR: 1.15; (95% CI 1.13–1.17); (P = 1.91 × 10^–76^)], were both associated with an increased risk of having had a MI event at baseline (Fig. 2). Conversely, diligence [OR: 0.80; (95% CI 0.78–0.82); (P = 1.51 × 10^–83^)], sociability [OR: 0.81; (95% CI 0.79–0.83); (P = 9.27 × 10^–76^)], and warmth [OR: 0.88; (95% CI 0.87–0.90); (P = 9.47 × 10^–58^)], were associated with a lower prevalence of MI events. Curiosity, the proxy for openness, was not significantly associated with prevalent MI at baseline [OR: 1.01; (95% CI 0.98–1.03); (P = 0.721)].

The same trends for prevalent MI prevailed when analyses were additionally adjusted for health-related measures in Model II (Fig. 2). The odds of having a MI event at baseline were higher for neuroticism [OR: 1.05; (95% CI 1.04–1.05); (P = 9.73 × 10^–30^)] and nervousness [OR: 1.12; (95% CI 1.10–1.14); (P = 1.12 × 10^–46^)]. Diligence [OR: 0.84; (95% CI 0.82–0.86); (P = 1.11 × 10^–45^)], sociability [OR: 0.85; (95% CI 0.83–0.87); (P = 1.44 × 10^–44^)] and warmth [OR: 0.89; (95% CI 0.88–0.90); (P = 1.69 × 10^–45^)] were all associated with lower risks of prevalent MI. Similar to Model I, curiosity [OR: 1.00; (95% CI 0.97–1.03); (P = 0.891)] was not significantly associated with prevalent MI in Model II.

In the fully adjusted model (Model III), when lifestyle factors were taken into account in addition to demographic and health-related measures, nervousness [OR: 1.10; (95% CI 1.08–1.12); (P = 8.40 × 10^–27^)], inferred the greatest personality-related risk for prevalent MI, followed by neuroticism [OR: 1.04; (95% CI 1.03–1.05); (P = 5.03 × 10^–16^)] (Fig. 2). Diligence [OR: 0.87; (95% CI 0.84–0.89); (P = 1.05 × 10^–25^)], sociability [OR: 0.89; (95% CI 0.87–0.92); (P = 7.17 × 10^–16^)] and warmth [OR: 0.90; (95% CI 0.89–0.92); (P = 3.68 × 10^–27^)] associated with reduced risk for MI events at baseline. Similar to Model I and II, curiosity [OR: 1.02; (95% CI 0.99–1.05); (p = 0.285)] was not associated with prevalent MI in the fully adjusted model.

#### Sex-specific associations between personality trait proxies and prevalent MI

To investigate the potential differences among men and women with regard to associations between personality and prevalent MI, sex-stratified analyses were conducted for all models (Supplementary Fig. S1). In the fully adjusted model, nervousness was associated with slightly higher risk for prevalent MI in women [OR: 1.14; (95% CI: 1.10–1.19); (P = 3.29 × 10^–10^)], compared to men [OR: 1.09; (95% CI: 1.07–1.11); (p = 4.75 × 10^–19^)]. Neuroticism was associated with same risk for prevalent MI in both women [OR: 1.04; (95% CI: 1.02–1.06); (P = 7.05 × 10^–5^)] and men [OR: 1.04; (95% CI: 1.03–1.05); (P = 8.80 × 10^–13^)]. For women, diligence [OR: 0.81; (95% CI: 0.76–0.86); (P = 3.55 × 10^–11^)] and sociability [OR: 0.86; (95% CI: 0.80–0.91); (P = 1.24 × 10^–6^)] appeared to be associated with lower risk for MI at baseline compared to men (diligence [OR: 0.88; (95% CI: 0.85–0.91); (P = 5.36 × 10^–17^)] and sociability [OR: 0.90; (95% CI: 0.88–0.93); (P = 1.96 × 10^–11^)] ). Warmth showed similar associations with MI both in women [OR: 0.88; (95% CI: 0.85–0.92); (P = 8.04 × 10^–9^) and men [OR: 0.91; (95% CI: 0.89–0.93); (P = 3.05 × 10^–20^). Curiosity was not associated with prevalent MI in either women [OR: 1.03; (95% CI: 0.95–1.11); (P = 0.462)] or men [OR: 1.01; (95% CI: 0.98–1.05); (P = 0.446)] (Supplementary Fig. S1).

### Association of personality trait proxies with incident MI

**Figure 3:**
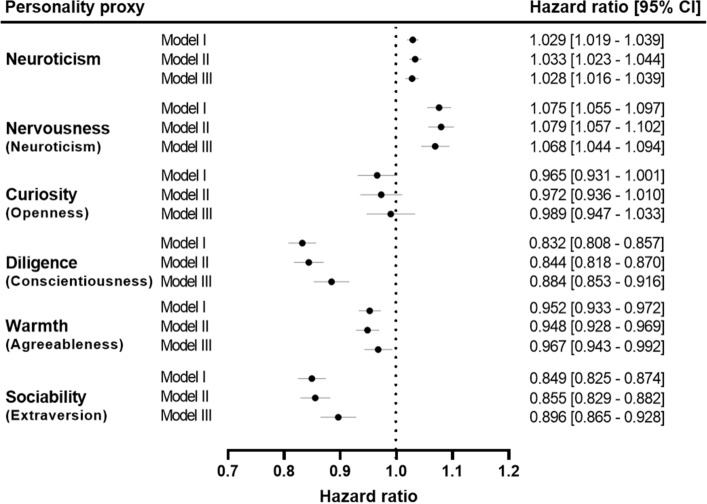
Association of neuroticism and personality trait proxies with incident myocardial infarction. Labels in small brackets represent original Big Five personality trait corresponding to each personality trait proxy. Model I: analyses were adjusted for demographic factors (sex, age, Townsend Deprivation Index and ethnic background). Model II: analyses were adjusted for health-related measures (body mass index, systolic blood pressure, diastolic blood pressure and diabetes) in addition to demographic factors. Model III (or fully adjusted model): analyses were adjusted for lifestyle factors (alcohol intake frequency, smoking status, and physical activity) in addition to demographic factors and health-related measures. The plot was created using GraphPad Prism (9.1.2).

The longitudinal association of personality trait proxies with MI are displayed in Fig. 3. Results from Model I showed increased risk of experiencing an MI event during the follow-up for neuroticism [HR: 1.03; (95% CI: 1.02–1.04); (P = 7.63 × 10^–9^)] and nervousness [HR: 1.08; (95% CI: 1.06–1.10); (P = 4.27 × 10^–13^)]. Similar to prevalent MI, diligence [HR: 0.83; (95% CI: 0.81–0.86); (P = 3.75 × 10^–34^)], sociability [HR: 0.85; (95% CI: 0.83–0.87); (P = 5.60 × 10^–28^)], and warmth [HR: 0.95; (95% CI: 0.93–0.97); (P = 3.60 × 10^–6^)], were associated with a lower incidence of MI events. Curiosity was not a significant predictor of MI [HR: 0.97; (95% CI: 0.93–1.00); (P = 0.055)].

After additionally adjusting the analyses for health-related measures in Model II, neuroticism [HR: 1.03; (95% CI: 1.02–1.04); (P = 4.45 × 10^–10^)] and nervousness [HR: 1.08; (95% CI: 1.06–1.10); (P = 6.13 × 10^–13^)] still accounted for significantly increased risks of incident MI. Diligence [HR: 0.84; (95% CI: 0.82–0.87); (P = 1.69 × 10^–26^)], sociability [HR: 0.86; (95% CI: 0.83–0.88); (P = 4.70 × 10^–23^)] and warmth [HR: 0.95; (95% CI: 0.93–0.97); (P = 1.62 × 10^–6^)] showed comparable protective associations towards developing MI as in Model I, while curiosity [HR: 0.97; (95% CI: 0.94–1.01); (P = 0.149)] showed no significant association with incident MI.

In accordance with the cross-sectional analysis, the fully adjusted model (Model III) showed that nervousness [HR: 1.07; (95% CI: 1.04–1.09); (P = 2.72 × 10^–8^)] and neuroticism [HR: 1.03; (95% CI: 1.02–1.04); (P = 2.52 × 10^–6^)] are associated with increased risk of incident MI event. Diligence [HR: 0.88; (95% CI: 0.85–0.92); (P = 1.43 × 10^–11^)], sociability [HR: 0.90; (95% CI: 0.87–0.93); (P = 1.49 × 10^–9^)] and warmth [HR: 0.97; (95% CI: 0.94–0.99); (P = 0.010)] were associated with lower risk for incident MI, while curiosity [HR: 0.99; (95% CI: 0.95–1.03); (P = 0.623)] showed no significant association.

#### Sex-specific associations between personality trait proxies and incident MI

Sex-stratification for the longitudinal associations between personality and MI was performed for all models (Supplementary Fig. S2). Nervousness was associated with a higher risk for incident MI in women [HR: 1.13; (95% CI: 1.08–1.19); (P = 9.90 × 10^–8^)] compared to men [HR: 1.05; (95% CI: 1.02–1.08); (P = 0.001]), and same association trend continue for neuroticism (women [HR: 1.05; (95% CI:1.03–1.07); (P = 3.28 × 10^–5^)] and men [HR: 1.02; (95% CI: 1.01–1.03); (P = 0.003)]. Sociability was associated with lower risk for incident MI in both women [HR: 0.83; (95% CI: 0.77–0.89); (P = 1.93 × 10^–7^)] and men [HR: 0.92; (95% CI: 0.88–0.96); (P = 7.39 × 10^–5^)] but the risk was slightly lower among women compared to men. Diligence was the most protective against MI in both men [HR: 0.89; (95% CI: 0.85–0.92); (P = 5.79 × 10^–9^ and women [HR: 0.89; (95% CI: 0.83–0.95); (P = 0.001)]. Warmth appeared to be protective against incident MI among women [HR: 0.94; (95% CI: 0.90–0.99); (P = 0.010)] but not among men [HR: 0.98; (95% CI: 0.95–1.01); (P = 0.141)] in the fully adjusted model. Curiosity was not a significantly associated with the risk of incident MI in women [HR: 0.94; (95% CI: 0.86–1.03); (P = 0.172)] or men [HR: 1.01; (95% CI: 0.96–1.06); (P = 0.807)] (Supplementary Fig. S2).

## Discussion

To our knowledge, this study is the largest to date to highlight the effect of personality on the risk of developing MI. By using the phenotypically rich UKB resource, we were able to identify proxies that could mimic the Big Five personality traits and investigate their impact on cardiovascular health. Participants with diligent and sociable personalities were less likely to experience myocardial infarction, while risk was particularly higher among participants with a nervous personality. Adjustment for covariates covering demographics, health and lifestyle did not substantially alter these associations, suggesting the role of behavioural dispositions in MI.

Personality is a strong determinant for several health-related behaviours like alcohol consumption^36^, sleep habits^37^, and physical exercise^38^. Such behaviours are considered to be modifiable risk factors for CVDs^4^. Thus, it is plausible that personality traits themselves confer heightened or lowered risks of developing cardiovascular complications. By grouping key questions regarding psychological and social factors to reflect the Big Five personality dimensions, the current study was able to replicate previous findings of how personality may mediate the occurrence of CVDs. In line with studies linking neuroticism and Type D personalities to adverse cardiac outcomes, nervousness was a significant risk factor for prevalent and incident MI^15,39^. The common denominator across these personality descriptions is the experience of strong negative affect and social inhibition. This may physically manifest as heightened cortisol spikes in response to acute stress, a disrupted hypothalamus–pituitary–adrenal (HPA) axis and coronary artery calcification^40–42^. In this manner, chronic hostile and socially vulnerable characteristics may catalyse the risk of CVD^43^. Although nervousness had an MI prevalence rate of 1.10 (CI: 1.08–1.12) and incident rate of 1.07 (CI: 1.04–1.09), it is important to note that smoking had a 1.7-fold increased risk, men had a threefold higher risk, while diabetic participants had a nearly fivefold increased risk for MI events (Supplementary Table S2). Therefore, nervousness plays a minor, albeit statistically significant, role in the physiopathology of MI.

Just as certain dispositions may be adverse for cardiovascular health, those encompassing stronger positive affect have been found to be cardio protective^2^. The longitudinal analysis highlighted high diligence as the most protective personality trait against MI, which is supported by Jokela et al.’s (2014) pooled results from three large-scale prospective cohort studies^15^. Diligence, the proxy for conscientiousness, is characterised by features such as self-discipline, cautiousness and persistence^44^. Diligent individuals may be more likely to exercise, adhere to doctor’s recommendations, and be less inclined to engage in dangerous behaviours^38,45^. Similar to diligence, sociability, mimicking extraversion, was also associated with lower risks of prevalent and incident MI in the fully adjusted models. The claims of extraversion influencing CVD are more diverse in the existing literature, with no significant contributing or protective role detected by Nakaya et al. (2005) and Batty et al. (2016)^20,21^.

In contrast, Otonari and colleagues (2021) found that extraverted men had higher CVD risk scores, but this trend was not seen among women^16^. By sex-stratifying the analysis, this study also detected slight differences for sociability in men and women. Alike Otonari et al. (2021), sociable men had higher prevalence and incidence hazard ratios for MI compared to sociable women. However, as the MI risk ratios for both genders were below 1, sociability was a significant protective trait in the UKB cohort. The discrepancy between the results could possibly be explained by the large disparity between the number of smokers in their male (31.5% currently smoking) and female (6.3% currently smoking) samples^16^. Even though the current sex-specific analysis highlighted how nervousness may constitute a greater risk for MI in women compared to men, the differences in associations are minimal. Additional evidence is required to address potential differential interactions between personality and gender, which could be useful in recognising gender-specific risk factors and preventing the development of cardiovascular problems.

In addition to diligence and sociability, another potentially protective trait was warmth. Although not directly targeted in the key questions for warmth (Table 1), the closely related trait optimism, may be contributing to the slight protection from MI^46^. In contrast to Lee et al. (2014), curiosity, the proxy for openness, was not a significant predictor for risk or protection against MI prevalence or incidence in any of the adjusted models^17^. However, the relatively small sample size of 661 participants, 18 incident MI events during the ten-year follow-up, and the inclusion of participants with personality and psychiatric disorders may underlie the differences in results with the current study^17^.

The main strengths of our study included a large sample size to ensure high statistical power, detailed information on covariates to control for potential confounding factors and most importantly the ability to identify the potential proxies to mimic Big Five personality traits. Moreover, by using our personality trait proxies, we were able to replicate and clarify the previous associations with MI. The only Big Five personality trait already available in the UKB is the validated neuroticism score, derived from the EPQ-N, which has successfully been used in prior UKB studies linking the trait to BMI, obesity, cognition and job satisfaction^47,48^. We used neuroticism to evaluate its proxy, nervousness, that followed the same trend. Future work would benefit from determining the convergent validity of the developed proxies with other Big Five measures. This would open up the possibility to conduct extended analyses linking the proxies to other diseases and disorders which could help in identifying at risk individuals and developing personalized interventions. Moreover, we could exclude participants with diagnosed personality and psychiatric disorders to ensure that the calculated risks are attributed to personality itself, and not to mental illness. The reliability of our findings is further strengthened by the fact that MI cases were identified by using official hospital records and death registries during the ~ 7-years of follow-up. Lastly, the consistency of the HRs calculated for each personality proxy using multiple models adjusting for potential confounding factors decreases the likelihood of by chance findings.

There are certain limitations of the study that should be acknowledged. A main limitation of the study, which accompanies the development and implementation of any new measure, is the use of un-validated personality traits. Thus, it is important to emphasise that the proxies only mimic the Big Five traits (extraversion, agreeableness, conscientiousness, openness and neuroticism) and the results should be interpreted with caution. Regardless, the framework for the current proxies: sociability, warmth, diligence, curiosity, and nervousness, follows the framework for established and frequently used personality tests. For example, the Midlife Development Inventory (MIDI) Personality Scales uses self-ratings of 30 adjectives to determine where the participants lie on the Big Five dimensions and the additional trait Agency^49^. Each trait is determined using four to seven adjectives, which is comparable to the four to five key questions used to create the current proxies. Despite this, more elaborate personality inventories with subscales, e.g., the revised NEO Personality Inventory, could be beneficial for identifying which specific facets infer elevated risks of CVDs^50^.

To conclude, diligent and sociable personality traits reduce the risk of prevalent and incident MI, while nervousness increases the risk of MI. The novel personality proxies show great potential to be applied to other disease areas within the large-scale UKB population to increasingly characterise how personality can both predispose and protect against specific illnesses. This will in turn help to develop individualised and personality-focused prevention strategies, which are particularly needed to reverse the increasing global burden of CVDs.

## Supplementary Information

Supplementary Information.

## Data Availability

The data used in this current study is available from the UK Biobank data resources. Permissions are required in order to gain access to the UK Biobank data resources, subject to successful registration and application process. Further information can be found on the UK Biobank website (https://www.ukbiobank.ac.uk/).
