# Supplementary material for: The influence of personality on the risk of myocardial infarction in UK Biobank cohort
Source: Sci Rep. 2022 Apr 25;12:6706. doi: 10.1038/s41598-022-10573-6 (PMC9038723; doi:10.1038/s41598-022-10573-6)
Supplement: Supplementary file 1 — Supplementary Information. [file 41598_2022_10573_MOESM1_ESM.docx]

**The influence of personality on the risk of myocardial infarction in UK Biobank cohort**

Amelia Dahlén, Maud Miguet, Helgi B. Schiöth and Gull Rukh

Supplementary material

| **Supplementary Table S1. ICD-10 codes for excluded diseases and disorders in UK Biobank participant sample** | |
| --- | --- |
| **Exclusion category** | **ICD-10 codes** |
| Personality disorders | Organic personality disorder´: 'F071', 'F072', 'F078', 'F079', 'F09'  Mental and behavioural disorders due to use of alcohol: 'F100', 'F101', 'F102', 'F103', 'F104', 'F105', 'F106', 'F108', 'F109'  Mental and behavioural disorders due to use of opioids: 'F110', 'F111', 'F112', 'F113', 'F114', 'F115', 'F116', 'F117', 'F118', 'F118', 'F119'  Mental and behavioural disorders due to use of cannabinoids: 'F120', 'F121', 'F122', 'F123', 'F124', 'F125', 'F126', 'F127', 'F128', 'F129'  Mental and behavioural disorders due to use of sedatives or hypnotics: 'F130', 'F131', 'F132', 'F133', 'F134', 'F135', 'F136', 'F137', 'F138', 'F139'  Mental and behavioural disorders due to use of cocaine: 'F140', 'F141', 'F142', 'F143', 'F144', 'F145', 'F146', 'F147', 'F148', 'F149'  Mental and behavioural disorders due to use of other stimulants, including caffeine: 'F150', 'F151', 'F152', 'F153', 'F154', 'F155', 'F156', 'F157', 'F158', 'F159'  Mental and behavioural disorders due to use of hallucinogens: 'F160', 'F161', 'F162', 'F163', 'F164', 'F165', 'F166', 'F167', 'F168', 'F169'  Mental and behavioural disorders due to use of tobacco: 'F170', 'F171', 'F172', 'F173', 'F174', 'F175', 'F176', 'F177', 'F178', 'F179'  Mental and behavioural disorders due to use of volatile solvents: 'F180', 'F181', 'F182', 'F183', 'F184', 'F185', 'F186', 'F187', 'F189'  Mental and behavioural disorders due to multiple drug use and use of other psychoactive substances: 'F190', 'F191', 'F192', 'F193', 'F194', 'F195', 'F196', 'F197', 'F198', 'F199'  Specific personality disorders: 'F60', 'F601', 'F602', 'F603', 'F604', 'F605', 'F606', 'F607', 'F608', 'F609', 'F61'  Enduring personality changes, not attributable to brain damage and disease: 'F620', 'F621', 'F628', 'F629'  Habit and impulse disorders: 'F630', 'F631', 'F632', 'F633', 'F638', 'F639'  Gender identity disorders: 'F640', 'F641', 'F642', 'F648', 'F649'  Disorders of sexual preference: 'F650', 'F651', 'F652', 'F653', 'F654', 'F655', 'F656', 'F658', 'F659'  Psychological and behavioural disorders associated with sexual development and orientation: 'F660' 'F661', 'F662', 'F668', 'F669'  Other disorders of adult personality and behaviour: 'F680', 'F681', F688'  Unspecified disorder of adult personality and behaviour: 'F69' |
| Psychiatric disorders | Schizophrenia: 'F200', 'F201', 'F202', 'F203', 'F204', 'F205', 'F206', 'F207', 'F208', 'F209'  Schizotypal disorder: 'F21'  Persistent delusional disorders: 'F220', 'F228', 'F229'  Acute and transient psychotic disorders: 'F230', 'F231', 'F232', 'F233', 'F238', 'F239'  Induced delusional disorder: 'F24'  Schizoaffective disorders: 'F250', 'F251', 'F252', 'F258', 'F259', 'F28', 'F29'  Manic episode: 'F300', 'F301', 'F302', 'F308', 'F309'  Bipolar affective disorder: 'F310', 'F311', 'F312', 'F313', 'F314', 'F315', 'F316', 'F317', 'F318', 'F319'  Depressive episode: 'F320', 'F321', 'F322', 'F323', 'F328', 'F329'  Recurrent depressive disorder: 'F330', 'F331', 'F332', 'F333', 'F334', 'F338', 'F339'  Persistent mood [affective] disorders: 'F340', 'F341', 'F348', 'F349'  Other mood [affective] disorders: 'F380', 'F381', 'F388', 'F39'  Phobic anxiety disorders: 'F400', 'F401', 'F402', 'F408', 'F409'  Other anxiety disorders: 'F410', 'F411', 'F412', 'F413', 'F418', 'F419'  Obsessive-compulsive disorder: 'F420', 'F421', 'F422', 'F428', 'F429'  Reaction to severe stress, and adjustment disorders: 'F430', 'F431', 'F432', 'F438', 'F439'  Dissociative [conversion] disorders: 'F440', 'F441', 'F442', 'F443', 'F444', 'F445', 'F446', 'F447', 'F448', 'F449'  Somatoform disorders: 'F450', 'F451', 'F452', 'F453', 'F454', 'F458', 'F459'  Other neurotic disorders: 'F480', 'F481', 'F488', 'F489'  Eating disorders: 'F500', 'F501', 'F502', 'F503', 'F504', 'F505', 'F508', 'F509' |
| Stroke | 'I600', 'I601', 'I602', 'I603', 'I604', 'I605', 'I606', 'I607', 'I608', 'I609', 'I610', 'I611', 'I612', 'I613', 'I614', 'I615', 'I616', 'I618', 'I619', 'I619', 'I630', 'I631', 'I632', 'I633', 'I634', 'I635', 'I636', 'I638', 'I639', 'I64', |
| Angina pectoris | 'I200', 'I201', 'I208', 'I209' |

| **Supplementary Table S2. Associations of covariates with prevalent and incident myocardial infarction (MI)** | | | | | | | | |
| --- | --- | --- | --- | --- | --- | --- | --- | --- |
|  | **Prevalence of MI** | | | | **Incidence of MI** | | | |
|  | **OR** | **95% CI** | | **Sig.** | **HR** | **95% CI** | | **Sig.** |
|  |  | Lower | Upper |  |  | Lower | Upper |  |
| **Demographic characteristics** | | | | | | | | |
| Age | 1.109 | 1.105 | 1.113 | 0.000 | 1.073 | 1.069 | 1.077 | 6.953 × 10^-252^ |
| Sex | 5.011 | 4.753 | 5.283 | 0.000 | 3.217 | 3.022 | 3.426 | 2.999 × 10^-292^ |
| Townsend deprivation index | 1.070 | 1.063 | 1.076 | 8.794 × 10^-96^ | 1.042 | 1.033 | 1.051 | 2.155 × 10^-20^ |
| Ethnicity | 1.063 | 0.969 | 1.168 | 0.197 | 1.031 | 0.910 | 1.168 | 0.637 |
| **Health-related measurements** | | | | | | | | |
| Systolic blood pressure | 0.998 | 0.997 | 0.999 | 0.003 | 1.020 | 1.019 | 1.021 | 3.428 × 10^-179^ |
| Diastolic blood pressure | 0.968 | 0.966 | 0.970 | 2.435 × 10^-191^ | 1.023 | 1.020 | 1.026 | 1.698 × 10^-61^ |
| Body mass index | 1.075 | 1.071 | 1.079 | 0.000 | 1.050 | 1.045 | 1.055 | 4.809 × 10^-78^ |
| Diabetes | 2.917 | 2.342 | 3.632 | 1.138 × 10^-21^ | 4.908 | 3.918 | 6.147 | 1.301 × 10^-43^ |
| **Lifestyle factors** | | | | | | | | |
| Physical activity (MET score) | 1.000 | 1.000 | 1.000 | 0.003 | 1.000 | 1.000 | 1.000 | 0.022 |
| Smoking | 1.703 | 1.656 | 1.753 | 1.537 × 10^-293^ | 1.681 | 1.619 | 1.745 | 3.341 × 10^-160^ |
| Alcohol intake frequency | 0.937 | 0.924 | 0.949 | 3.987 × 10^-21^ | 0.936 | 0.919 | 0.953 | 6.293 × 10^-13^ |


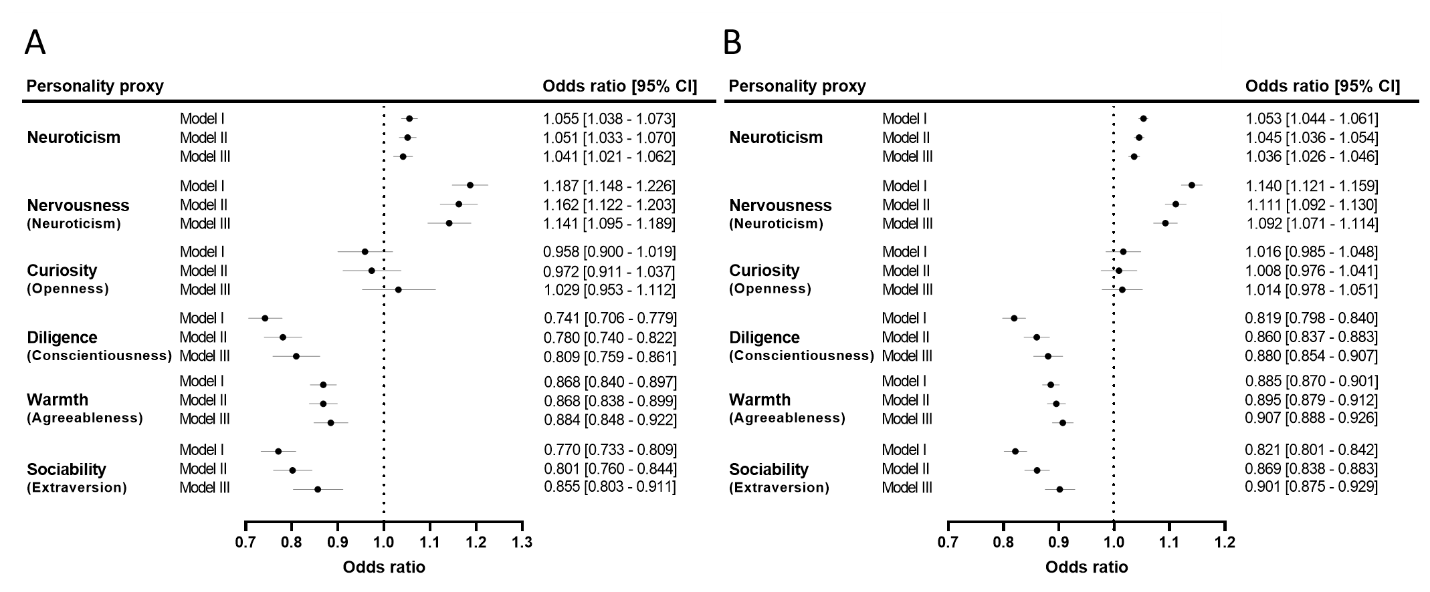


**Figure S1.** Association of neuroticism and personality trait proxies with prevalent myocardial infarction in women **(A)** and men **(B).** Labels in small brackets represent original Big Five personality trait corrresponding to each personality trait proxy. Model I: analyses were adjusted for demographic factors (age, townsend deprivation index and ethnic background). Model II: analyses were adjusted for health-related measures (body mass index, systolic blood pressure, diastolic blood pressure and diabetes) in addition to demographic factors. Model III (or fully adjusted model): analyses were adjusted for lifestyle factors (alcohol intake frequency, smoking status, and physical activity) in addition to demographic factors and health-related measures.


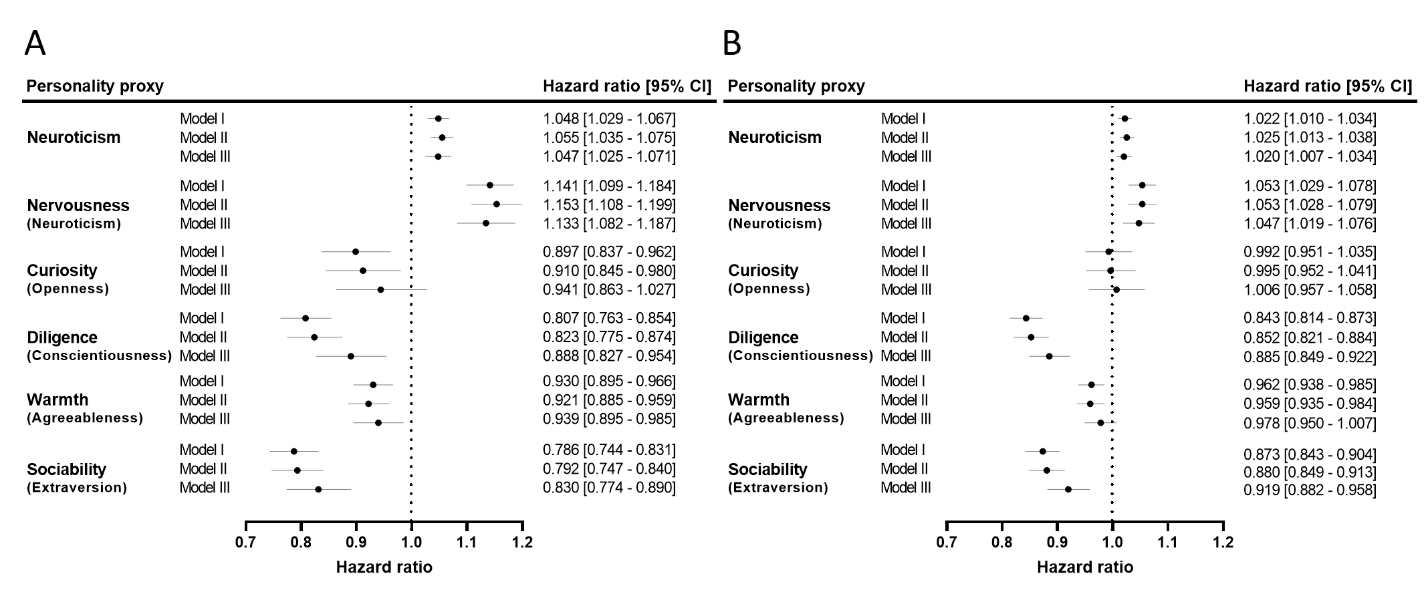


**Figure S2.** Association of neuroticism and personality trait proxies with incident myocardial infarction in women **(A)** and men **(B).** Labels in small brackets represent original Big Five personality trait corrresponding to each personality trait proxy. Model I: analyses were adjusted for demographic factors (age, townsend deprivation index and ethnic background). Model II: analyses were adjusted for health-related measures (body mass index, systolic blood pressure, diastolic blood pressure and diabetes) in addition to demographic factors. Model III (or fully adjusted model): analyses were adjusted for lifestyle factors (alcohol intake frequency, smoking status, and physical activity) in addition to demographic factors and health-related measures.
